# Phytochemical Profile and Antioxidant and Anti-Inflammatory Properties of *Larrea cuneifolia*, a Medicinal Plant of Argentina, Obtained by Micropropagation, Acclimatized, and Established in the Field, Compared with Wild Plants

**DOI:** 10.3390/plants15213323

**Published:** 2026-10-27

**Authors:** Camila Pedraza Kobak, María Celeste Barrera, Fátima Carolina Danert, Iris Catiana Zampini, María Inés Isla

**Affiliations:** 1 Instituto de Bioprospección y Fisiología Vegetal (INBIOFIV-CONICET-UNT), San Miguel de Tucumán T4000CBG, Tucumán, Argentina; camileno@gmail.com (C.P.K.); mariacelestebarrera92@gmail.com (M.C.B.); fcdanert@gmail.com (F.C.D.); zampini@csnat.unt.edu.ar (I.C.Z.); 2 Facultad de Ciencias Naturales e IML, Universidad Nacional de Tucumán (UNT), Miguel Lillo 205, San Miguel De Tucumán T4000CBG, Tucumán, Argentina

**Keywords:** *Larrea cuneifolia*, medicinal plant, micropropagation, metabolomics, phytochemical fingerprint, nordihydroguaiaretic acid, antioxidant and anti-inflammatory activities, sustainable production

## Abstract

*Larrea cuneifolia* Cav. (“jarilla macho”) is a medicinal shrub native to arid regions of Argentina with recognized antioxidant, anti-inflammatory, antitumoral, antibacterial, and antifungal properties. However, its slow growth and dependence on wild harvesting limit its sustainable utilization. This study developed an efficient protocol for *in vitro* propagation, greenhouse acclimatization, and field establishment of *L. cuneifolia* and evaluated the phytochemical profile and biological activities of micropropagated plants in comparison with those of wild individuals. Seeds were germinated under aseptic conditions, and nodal explants were cultured on media supplemented with different plant growth regulators. Indole-3-acetic acid (IAA, 1 mg/L) provided the best overall response, promoting leaf production, nodal development, and rooting. Plantlets showed 95% survival during greenhouse acclimatization and 80% survival after field establishment in the Valles Calchaquíes, Argentina. Acclimatization was accompanied by increased accumulation of phenolic compounds and nordihydroguaiaretic acid (NDGA), the principal lignan of the genus *Larrea*. High-performance liquid chromatography–heated electrospray ionization–Orbitrap high-resolution tandem mass spectrometry (HPLC–HESI–Orbitrap–HR MS/MS) analysis showed that propagated plants retained the characteristic phytochemical profile of wild *L. cuneifolia*, including its lignan-rich epicuticular resin. Despite differences in metabolite abundance and diversity, antioxidant and anti-inflammatory activities remained comparable to those of wild plants. These results demonstrate that micropropagated *L. cuneifolia* plants retain their phytochemical identity, NDGA accumulation capacity, biological activity, and metabolic plasticity after acclimatization and field establishment. The protocol therefore provides a promising strategy for *ex situ* conservation and sustainable production of *L. cuneifolia* biomass for potential pharmaceutical and cosmetic applications while reducing harvesting pressure on natural populations.

## 1. Introduction

Argentina has a relatively high diversity of plant species. However, uncontrolled harvesting, urbanization, climate change, and ecosystem degradation are increasingly threatening native plant populations and contributing to the loss of plant genetic resources. Consequently, numerous endemic species have been classified as threatened and included in the national Red List of Endemic Plants of Argentina [1]. Many of these species are valuable for their ornamental, medicinal, cosmetic, and/or aromatic properties [2]. The increasing threats to these plant resources have emphasized the need to develop effective conservation strategies. Cultivation under controlled conditions represents an important approach for *ex situ* conservation, while also providing plant material for sustainable use and potentially supporting *in situ* conservation efforts. In this context, *in vitro* culture is a valuable tool for the propagation of selected plant material for conservation and cultivation purposes [3].

*Larrea cuneifolia* Cav., a native medicinal plant from Argentina, was included in the Red List of Endemic Plants of Argentina [1]. It is a xerophytic shrub with perennial leaves and a woody stem, reaching up to 2 m in height. It is widely distributed throughout the Monte Biogeographic Province, from northern Argentina to northern Patagonia, and occurs at elevations of up to 3000 m above sea level (a.s.l.). In northwestern Argentina, indigenous communities of the Valles Calchaquíes have a long history of using *L. cuneifolia* for medicinal purposes, particularly for respiratory, musculoskeletal and skin disorders [4,5,6]. *L. cuneifolia* is recognized for its anti-inflammatory, antirheumatic, hypotensive, rubefacient, diaphoretic, febrifuge, oxytocic, emmenagogue, odontalgic and antitussive properties, as well as its antifungal properties [4,5,6,7,8]. In this sense, several studies have documented its antimicrobial activity against multi-resistant Gram-positive and Gram-negative bacteria, as well as antifungal against phytopathogenic filamentous fungi and yeasts, larvicidal activity, antioxidant, anti-inflammatory and antitumoral [9,10,11,12,13,14,15,16,17,18,19] properties. In Argentina, herbal medicines with a documented history of traditional use may be registered as “Herbal Medicines of Traditional Use” under the regulatory framework established by the Administración Nacional de Medicamentos, Alimentos y Tecnología Médica (ANMAT), Disposition No. 5418/2015 [20]. Given its long-standing traditional medicinal use, *L. cuneifolia* represents a potential source of plant material for the development of herbal products within this regulatory framework. More recently, its capacity to accumulate metals and tolerate highly contaminated soils has also been reported [21]. Despite its ecological and pharmacological importance, the sustainable use of *L. cuneifolia* requires strategies that reduce dependence on harvesting from natural populations.

Seed dormancy, low seedling emergence (approximately 20% after one year), and the long period required to obtain mature plants make the conventional cultivation of *L. cuneifolia* challenging [22,23]. Although information on the time required for *L. cuneifolia* to reach reproductive maturity is currently lacking, the related species *L. tridentata* has been reported to begin fruiting at 4–6 years of age and to reach full reproductive maturity at 8–13 years [24]. These limitations highlight the need for alternative propagation strategies. Micropropagation may provide an efficient approach for the conservation and multiplication of *L. cuneifolia*, while also providing plant material for sustainable pharmaceutical and cosmetic applications. However, to our knowledge, a comprehensive protocol integrating *in vitro* propagation, greenhouse acclimatization, and subsequent field establishment has not yet been developed for this species. Therefore, this study aimed to establish an effective protocol for the *in vitro* propagation, acclimatization, and field establishment of *L. cuneifolia*, and to evaluate whether propagated plants retain the phytochemical characteristics and biological activities of wild plants. This approach could contribute to the sustainable production of bioactive plant material while reducing harvesting pressure on natural populations.

## 2. Results and Discussion

### 2.1. Germination

A germination capacity of 81% and a mean germination time of 2.65 days were recorded. Seed dormancy is a common adaptive trait in wild species, preventing germination even under favorable environmental conditions and frequently resulting in delayed and asynchronous germination [22,23,25,26]. Previous studies have reported physical dormancy in species of the genus *Larrea*, and several pre-germinative treatments, particularly mechanical scarification, have been recommended to enhance germination [27,28]. However, under the conditions of the present study, seeds exhibited high germination without any pre-germinative treatment, suggesting that the seed exhibited only a negligible level of dormancy. Differences among studies may be related to seed provenance, environmental conditions during seed development, seed maturity, storage conditions, or natural variation in dormancy intensity among populations.

### 2.2. In Vitro Induction and Multiplication and Rooting of Shoots

The Valles Calchaquíes region is notable for the presence of endangered endemic species that serve as potential sources of metabolites with pharmaceutical and cosmetic applications. In this context, plant biotechnology through micropropagation or *in vitro* culture offers a solution for conserving and propagating these threatened plants. Shoot cultures of *L. cuneifolia* were successfully established from binodal segments of 8-week-old *in vitro* grown seedlings derived from wild plant seeds. Explant response was strongly influenced by the type and concentration of plant growth regulators, as well as by the duration of the culture period. Although treatments with KIN 0.25 mg/L and IAA 1.5 mg/L yielded numerically higher rooting percentages (88% and 80%, respectively) than 1 mg/L IAA (62.5%), pairwise comparisons revealed no significant differences. In contrast, 1 mg/L IAA induced the highest mean number of leaves (14.44 ± 5.12) and nodes (7.38 ± 3.56) per explant, after 60 days of culture (Table 1). Since this species exhibits spontaneous rhizogenesis during multiplication, a separate rooting phase was not required. Among the evaluated cytokinins, kinetin performed best, whereas BAP treatments generally showed poorest results (except at the highest concentration), and the combined IBA + BAP treatment under performed compared to the control. Consequently, 1 mg/L IAA provided the most favorable balance between vegetative vigor and root formation, establishing it as the optimal treatment for subsequent *ex vitro* acclimatization.

This integration of parameters is crucial for large-scale *in vitro* propagation. Because nodal multiplication determines the yield of propagules throughout subcultures, 1 mg/L of IAA effectively enhances overall vegetative development. This highlights that the effectiveness of the protocol must be evaluated based on coordinated growth rather than root induction alone, a principle that has been emphasized in other species from arid region of South American, such as *Zuccagnia punctata*, where superior seedling quality takes precedence over mere root maximization [29].

Principal component analysis (PCA) provided an integrated view of the relationships between micropropagation treatments and morphogenic responses (Figure 1). The first two principal components accounted for 62.3% of the total variation, with PC1 and PC2 explaining 41.2% and 21.1%, respectively. IAA at 1 mg/L was positioned on the positive side of PC1 and was associated with the vectors representing the number of leaves and nodes at 60 days, consistent with the high values observed for these variables in the univariate analysis. In contrast, treatments such as NAA at 1 mg/L and BAP at 2 and 4 mg/L were positioned toward positive PC2 values and were more closely associated with callus formation. Overall, the PCA supports the distinct morphogenic response induced by IAA at 1 mg/L, characterized primarily by enhanced vegetative development rather than callus formation.

### 2.3. Ex Vitro Acclimatization

The *ex vitro* acclimatization phase revealed a high adaptive capacity in the micropropagated plants. During the first acclimatization phase, conducted under temperature-controlled conditions in an acclimatization chamber (greenhouse), a survival rate of 95% was recorded (Figure 2), indicating a successful transition from *in vitro* conditions to a protected *ex vitro* environment. The high survival rate during acclimatization and the development of vigorous root systems demonstrates the efficacy of the established protocol. The acclimatized plants exhibited a normal, healthy morphological appearance, with no visible abnormalities.

Subsequently, during the second acclimatization stage, which involved transplanting and establishment of plants under field conditions in Ampimpa, located at 1960 m a.s.l. in Valles Calchaquies (Argentina), an 80% survival rate was observed. Although this represented a slight decrease compared with the first stage, the results reflect the plants’ strong capacity to establish themselves under more restrictive and variable environmental conditions (Figure 2). The acclimatization protocol proved effective in promoting the gradual adaptation of micropropagated plants, ensuring high survival rate under *ex vitro* and field conditions. Similar results were obtained by *in vitro* culture of *Senecio nutans*, an endemic plant of the Atacama Desert Andean Region [30].

### 2.4. Phytochemical Characterization of In Vitro Micropropagated, Ex Vitro-Acclimatized, and Wild Larrea cuneifolia Plants

#### 2.4.1. Total Phenolic and Flavonoids Content

The changes in total phenolic and flavonoid contents throughout the different stages of acclimatization and field establishment reveal a dynamic pattern of specialized metabolism in *L. cuneifolia*. Total phenolic content, as estimated by the Folin–Ciocalteu assay, increased progressively from 2440 µg GAE/mL in two-month-old *in vitro* plants (MP-2) to 3140, 7020, and 8790 µg GAE/mL after 8, 11, and 15 months under greenhouse conditions, respectively (Table 2). This progressive accumulation may reflect the physiological adjustment of plants during acclimatization from the highly controlled *in vitro* environment to greenhouse conditions, where they are exposed to greater environmental variability, including changes in irradiance, temperature, and water availability. Phenolic compounds are an important component of plant antioxidant and defense systems, and changes in their accumulation have been widely associated with plant responses to environmental conditions and abiotic stress [31,32,33].

Interestingly, after transferring to field conditions, total phenolic content decreased to 7370 µg GAE/mL (MP-2+11G+4F), suggesting that the high accumulation observed during greenhouse acclimatization is not necessarily maintained after establishment under natural conditions. Rather than representing a continuous increase with plant age, these results suggest that phenolic accumulation is dynamic and may depend on the developmental and environmental conditions experienced by the plants. Consistent with this interpretation, young and adult wild plants showed total phenolic compounds content of 7190 and 7800 µg GAE/mL, respectively, which were relatively high but lower than the maximum reached by plants maintained for 15 months under greenhouse conditions.

A particularly interesting pattern was observed for the phenolic compounds recovered from the chloroform-soluble epicuticular resin. Their contribution to total leaf phenolic content increased from 28.9% after 8 months under greenhouse conditions to approximately 49–51% after 11–15 months, whereas it decreased to 40.9% after transfer to field conditions. In contrast, only 18.4% and 18.1% of total phenolics were recovered in the epicuticular resin of young and adult wild plants, respectively. Thus, although wild plants maintained relatively high total phenolic contents, a considerably smaller proportion was associated with the leaf surface resin. This difference suggests that acclimatization affects not only the total accumulation of phenolic compounds but also their relative distribution between the epicuticular resin and the remaining leaf fractions. The high proportion of surface-associated phenolics observed during greenhouse acclimatization may represent a transient metabolic response associated with the transition from *in vitro* to *ex vitro* conditions. Once *Larrea* species become established under field conditions, the relative allocation of phenolic compounds to the surface resin may change toward the pattern observed in naturally growing plants. However, since environmental variables were not independently controlled in the present study, the contribution of individual factors cannot be established from these results.

Flavonoids showed a related but not identical pattern. Total flavonoid content increased during the initial greenhouse acclimatization stage, reaching its highest value after 8 months, and subsequently decreased at 11 and 15 months and after field transfer. In contrast, the proportion of flavonoids recovered in the epicuticular resin was particularly high after 11 and 15 months under greenhouse conditions (approximately 68% of total flavonoids) and decreased markedly after field transfer and in wild plants. Young and adult wild plants showed only approximately 10–13% of their total flavonoids in the surface resin. The parallel reduction in the relative contribution of surface-associated phenolics and flavonoids from greenhouse-acclimatized to field-established and wild plants support the idea that the chemical composition and relative contribution of the epicuticular resin are highly dynamic during plant establishment. Furthermore, the increase in total phenolics accompanied by a decrease in total flavonoids at the later stages of greenhouse acclimatization suggests a change in the relative contribution of different classes of phenolic metabolites.

Non-flavonoid compounds, including lignans characteristic of the genus *Larrea*, may contribute to this pattern, although compound-specific chemical analyses would be necessary to confirm this hypothesis. Overall, these results indicate that micropropagated *L. cuneifolia* plants retain an active and plastic phenolic metabolism during acclimatization and field establishment, with changes occurring not only in total phenolic and flavonoid contents but also in their relative accumulation in the epicuticular resin.

#### 2.4.2. Recovery of NDGA Accumulation During Acclimatization and Field Establishment

Quantification of nordihydroguaiaretic acid (NDGA), the principal lignan of the genus *Larrea*, revealed marked changes throughout plant development and acclimatization (Table 3). Recently micropropagated plants (2 months *in vitro*) contained only low concentrations of NDGA (8.0 mg/g DW). However, NDGA content increased progressively during greenhouse acclimatization, reaching 66.8 and 101.2 mg/g DW after 8 and 15 months, respectively. Plants transferred to field conditions after 11 months in the greenhouse and maintained in the field for additional four months accumulated the highest NDGA concentration (112.2 mg/g DW), approximately 1.2-fold (18.6%) the value determined in young wild plants (94.6 mg/g DW).

This result indicates not only that the capacity for NDGA biosynthesis was fully retained after micropropagation and acclimatization, but also that the sequential transition from *in vitro* culture to greenhouse and subsequently to field conditions was associated with an enhanced accumulation of this lignan. In fact, NDGA content increased approximately 14-fold, rising from the initial MP-2 stage (8.0 mg/g DW) to field-established plants (112.2 mg/g DW). The higher concentration observed in MP-2+11G+4F plants compared with YWP may reflect the combined influence of developmental stage, prior acclimatization history, and environmental conditions on NDGA metabolism. Since these factors were not evaluated independently, the specific mechanism responsible for this difference cannot be established from the present study. Nevertheless, the results demonstrate that micropropagation does not restrict the capacity of *L. cuneifolia* to accumulate NDGA and that acclimatized plants can attain, and even exceed, the concentrations found in naturally growing young plants.

Plants grown under *in vitro* conditions develop under a highly controlled environment characterized by high humidity, low irradiance, continuous nutrient availability and the absence of significant abiotic stress. Under these conditions, the demand for defensive specialized metabolites is reduced. Conversely, greenhouse acclimatization and subsequent field establishment expose plants to increasing irradiance (ultraviolet radiation), water limitation and temperature fluctuations, factors known to activate the phenylpropanoid pathway and stimulate the biosynthesis of phenolic compounds, including lignans [31,34]. The increase in NDGA during acclimatization is also consistent with the natural plasticity of specialized metabolism previously described for *L. cuneifolia.* Barrera et al. (2025) [35] reported significant daily and seasonal variations in lignan and phenolic accumulation, demonstrating that environmental conditions regulate the production of these specialized metabolites. Therefore, the progressive recovery and subsequent increase in NDGA accumulation during greenhouse acclimatization and field establishment are consistent with the marked plasticity of specialized metabolism in *L. cuneifolia.*

NDGA has been associated with many of the biological properties attributed to *Larrea* genus including antioxidant, anti-inflammatory, antimicrobial, and cytoprotective activities [36,37,38]. Therefore, the recovery of NDGA accumulation following acclimatization demonstrates that the micropropagation process does not compromise the biosynthetic machinery responsible for lignan production. On the contrary, the progressive increase observed after greenhouse cultivation and field establishment indicates that micropropagated plants retain the capacity to synthesize this metabolite at levels comparable to, or even higher than, those found in naturally growing plants.

From an applied perspective, these findings are particularly relevant because they indicate that the micropropagation protocol developed for *L. cuneifolia* not only enables efficient clonal propagation but also preserves one of the principal phytochemical attributes responsible for the medicinal value of the species. This characteristic enhances the potential of micropropagation as a strategy for the sustainable production of high-quality plant material, while reducing harvesting pressure on natural populations.

#### 2.4.3. Comparative Phytochemical Profiling of Wild and Micropropagated *L. cuneifolia* Plants

Several studies have described the phytochemical composition of *L. cuneifolia* collected from different regions of Argentina, reporting phenolic acids, methylated flavonoids derived from quercetin, kaempferol and apigenin, together with lignans extracted using conventional organic and aqueous solvents and, more recently, green extraction systems such as natural deep eutectic solvents (NaDES) [6,9,11,35,39,40]. However, to the best of our knowledge, this is the first report describing the phytochemical profile of micropropagated *L. cuneifolia* plants following greenhouse acclimatization and field establishment.

HPLC–HESI–Orbitrap-HRMS/MS analysis revealed similarities and differences in the metabolomic profiles of field- established micropropagated and wild plants. A total of 29 metabolites were detected and tentatively identified in field-established micropropagated plants at 1960 m a.s.l., whereas 40 compounds were detected in wild plants (Table S1 and Figure S1). Wild plants showed greater metabolite diversity, particularly among features tentatively assigned to phenolic acids and flavonoids. Nevertheless, the two groups shared numerous metabolites, including compounds putatively assigned to phenolic acids, methylated flavonoids, stilbenes, and lignans, such as several NDGA-related compounds [32,36,37]. Lignans and methylated flavonoids have previously been described as chemotaxonomic markers of the genus *Larrea* [35,39,40].

These results indicate that, although the number and relative distribution of detected metabolites differed between micropropagated and wild plants, a substantial portion of the characteristic phytochemical profile of *L. cuneifolia* was retained after micropropagation, greenhouse acclimatization, and field establishment. Metabolite annotations were based on accurate precursor mass, proposed molecular formula, MS/MS fragmentation patterns, and comparison with previously reported data. Because authentic reference standards were not analyzed for these compounds under the same experimental conditions, these assignments should be considered tentative (Level 3 according to the confidence classification proposed by Schymanski et al. [41] rather than unequivocal structural identifications).

The phytochemical differences between both plant groups may be related to the contrasting developmental histories and environmental conditions experienced by them. Plants growing naturally in arid environments are exposed over prolonged periods to variations in water availability, solar radiation, and temperature, factors that have been associated with changes in phenylpropanoid metabolism and the accumulation of specialized metabolites [34,42]. In *L. cuneifolia*, Barrera et al. (2025) [35] previously demonstrated temporal plasticity in phenolic and lignan accumulation, with qualitative and quantitative variations occurring throughout the day and among seasons. Thus, the differences observed here between field-established micropropagated and wild plants may reflect, at least in part, differences in developmental stage, environmental history, and duration of exposure to natural conditions. However, because these factors were not independently evaluated in the present study, a direct causal relationship between specific environmental variables and the observed phytochemical differences cannot be established. Longer-term studies comparing plants of similar developmental stages under controlled environmental conditions would be necessary to determine the relative contributions of plant age, environmental exposure, and propagation history to the observed differences.

#### 2.4.4. Lignan Composition of the Epicuticular Resin and Its Ecological Significance

Analysis of leaf surface washings revealed a remarkably simple phytochemical profile compared with that of leaf extracts. In both wild and micropropagated plants, the epicuticular resin consisted almost exclusively of lignans, whereas flavonoids and phenolic acids were not detected (Table S1). This selective accumulation suggests that lignans are actively secreted to the leaf surface and constitute the principal chemical components of the protective resin covering *L. cuneifolia* leaves.

Similar observations have been reported in other species of the genus *Larrea.* In *L. tridentata*, NDGA and related lignans constitute approximately 80% of the resin phenolics and 40–50% of the total resin [36,38,43,44]. Likewise, lignans derived from NDGA together with ferulic acid derivatives have been described as the major constituents of the resinous exudates of *L. nitida* [45]. The ecological importance of these resinous exudates has been extensively discussed. The resin coating has been proposed to reduce cuticular transpiration, attenuate ultraviolet radiation, protect against herbivores and pathogens, and prevent oxidative degradation of exposed tissues through the strong antioxidant activity of lignans [38,45,46,47]. The presence of a lignan-rich resin in both micropropagated and wild plants demonstrates that the secretion of these compounds is maintained after *in vitro* propagation. This finding is particularly relevant because it indicates that micropropagation preserves not only the biosynthetic capacity for lignan production but also the physiological mechanisms responsible for their secretion onto the leaf surface. Since the epicuticular resin represents one of the principal adaptive traits of *Larrea* species inhabiting arid environments, maintaining this characteristic is likely to contribute to the successful establishment of micropropagated plants under natural field conditions.

### 2.5. Antioxidant and Anti-Inflammatory Activity

Reactive oxygen species (ROS), including superoxide anion (O_2_•^−^), hydroxyl radical (•OH) and hydrogen peroxide (H_2_O_2_), are continuously generated during aerobic metabolism in plants and animals. When their production exceeds the cell’s antioxidant capacity, oxidative stress can occur, leading to lipid peroxidation, protein oxidation and DNA damage. The specialized metabolites, particularly phenolic compounds such as phenolic acids, flavonoids and lignans produced by *L. cuneifolia* could contribute not only as antioxidant defense system in the plant but also could be used for the development of phytotherapic products for human or animal health [46,47,48,49,50,51]. Previous studies have demonstrated the antioxidant activity of extracts from wild *L. cuneifolia* obtained using different extraction solvents [6,9,12,16,19,35]. However, to date, the antioxidant potential of micropropagated plants following their acclimatization in a greenhouse and establishment in the field has not been investigated.

The ethanolic extracts obtained from leaves of micropropagated *L. cuneifolia* exhibited high ABTS cation radical scavenging activity (SC_50_ = 3.96 ± 0.08 μg GAE/mL). Comparable values were observed in plants acclimatized in the greenhouse for 11 months and subsequently established in the field (4.56 ± 0.12 μg GAE/mL), and in young wild plants (4.90 ± 0.09 μg GAE/mL). These results indicate that the antioxidant capacity is maintained throughout the micropropagation, acclimatization and field establishment processes, demonstrating that the propagation protocol preserves one of the principal biological properties of the species. The antioxidant activity could be ascribed to compounds common to extracts from micropropagated plants and wild plants growing in the Valles Calchaquíes with demonstrated antioxidant activity such as quinic acid and 3,4-dihydroxyhydrocinnamic acid, flavonoids derived from luteolin (eriodictyol) and quercetin (isorhamnetin, hispidulin and di-O-methylquercetin) as well as lignans [9,12,16,17,18,35,38,48,49,50,51].

Likewise, the plants reintroduced into the field showed hydrogen peroxide removal activity with values similar to those of young wild plants from the same region [17] (Table 4).

The extracts also showed strong inhibitory activity against xanthine oxidase (XO), an enzyme responsible for the oxidation of hypoxanthine and xanthine accompanied by the generation of superoxide radicals and uric acid. The accumulation of uric acid causes inflammation through production of leukotrienes. Thus, the inhibition of XO could be a strategy for the treatment of inflammatory processes.

Among the evaluated materials, greenhouse-acclimatized plants exhibited the highest inhibitory activity (IC_50_ = 3.41 μg GAE/mL), whereas field-acclimatized and wild plants showed comparable activities (IC_50_ values between 4.06 and 4.17 μg GAE/mL).

Interestingly, the antioxidant activities did not strictly parallel either total phenolic content or NDGA concentration. For example, plants with the highest NDGA levels did not always exhibit the strongest radical scavenging or xanthine oxidase inhibitory activities. This observation suggests that the antioxidant or anti-inflammatory potential of *L. cuneifolia* leaf extracts are determined not only by the concentration of individual metabolites but also by the qualitative composition of the phenolic fraction and the interactions among different bioactive compounds. Such synergistic or additive effects have been frequently reported for complex plant extracts, where flavonoids, phenolic acids and lignans collectively contribute to antioxidant and pro-inflammatory enzyme inhibitory activities [17].

## 3. Materials and Methods

### 3.1. Chemicals, Reagents and Materials

Ethanol 96% (analytical grade) and phenol ACS grade were purchased from Cicarelli (Santa Fe, Argentina). Commercial Folin–Ciocalteu reagent analytical grade was purchased from Anedra (Buenos Aires. Argentina), Aluminum chloride (AlCl_3_) 99% reagent grade, gallic acid 97.5–102.5%, titration, quercetin 95%, HPLC, 2,2′-azino-bis(3 ethylbenzothiazoline-6-sulfonic acid) diammonium salt (ABTS^•+^) ≥ 98%, HPLC, sucrose analytical grade, 4-aminoantipyrene ≥ 97%, horseradish peroxidase Type II, 150–250 U/mg, xanthine oxidase from bovine milk Grade III, ≥0.8 units/mg protein, xanthine 99.9%, 2,2′-azobis(2-methylpropionamidine)dihydrochloride (AAPH) 97%, Kinetin (KIN) ≥ 98% purity, H_2_O_2_, mass spectrometry grade and HPLC grade solvents and standards as nordihydroguaiaretic acid (NDGA) HPLC grade were obtained from Sigma Aldrich (St. Louis, MO, USA).

Gibberellic acid (GA_3_) ≥ 90% purity, 6-benzylaminopurine (BAP) ≥ 98% purity, indole-3-butyric acid (IBA) ≥ 90% purity, 1-naphthaleneacetic acid (NAA) ≥ 97% purity, indole-3-acetic acid (IAA) ≥ 97% purity and Murashige and Skoog basal medium were purchased from PhytoTech Labs (Lenexa, KS, USA). Bacteriological quality agar, microbiological grade; Britania (Buenos Aires, Argentina).

### 3.2. Plant Material, Taxonomic Identification, and Sampling

Mature fruits and aerial parts of wild plant material of *Larrea cuneifolia* Cav., were collected from naturally growing populations located in Ampimpa, Amaicha del Valle (Tafí del Valle Department, Tucumán, Argentina), at 1960 m a.s.l. (26°35′33″ S; 65°55′00″ W), in February 2022. The taxonomic identification of *Larrea cuneifolia* Cav. was performed by Dra. Soledad Cuello botanist from INBIOFIV, based on morphological characters/taxonomic key. A voucher specimen (voucher LIL 618.708) was deposited in the Fundación Miguel Lillo Herbarium, Argentina. The plant material was collected from 10 randomly selected adult plants within the same natural population. Subsequently, the fruits from the different source plants were pooled and stored in paper bags at room temperature (25 °C) for 1 week (Figure 3). Healthy, intact, and mature seeds, identified by their dark brown coloration, were manually extracted from the fruits for the *in vitro* propagation experiments.

For comparison of phytochemical composition and biological activity, wild plant leaves were collected during February 2022 from 5 independent adult plants from the same population. Micropropagated plants grown under greenhouse conditions for 8, 11, and 15 months were evaluated. For each experimental group, leaves from 5 independent plants were collected. Plant material from each one was processed separately for extraction.

A biological replicate was defined as plant material obtained from one independent plant and processed separately from collection through extraction and subsequent analyses. A total of *n* = 5 biological replicates were analyzed per experimental group. Each extract was analyzed in triplicate for phytochemical composition and biological activity; these repeated measurements were considered technical replicates and were not treated as independent biological replicates.

### 3.3. In Vitro Culture

#### 3.3.1. Seed Disinfection

For surface sterilization, seeds were first immersed in 70% (*v*/*v*) ethanol solution for one minute, followed by three rinses with sterile distilled water (two minutes per each one) at room temperature. Subsequently, seeds were immersed in a 15% (*v*/*v*) sodium hypochlorite solution (active chlorine 55 g/L) for 10 min with continuous agitation in an orbital shaker (50 rpm) under sterile conditions. Afterward, the seeds were rinsed three additional times with sterile distilled water (two minutes per wash) to minimize residual sodium hypochlorite before sowing. Finally, the aseptic seeds were cultured under *in vitro* conditions to obtain the explants. The germination power (GP) of seeds was determined

#### 3.3.2. Culture Medium Preparation

Half-strength Murashige and Skoog (1/2 MS) medium [52] was used as the basal medium (BM). The BM was supplemented with 30 g/L sucrose and 100 mg/L ascorbic acid (antioxidant) and 8 g/L agar was added. This medium was named culture medium (CM). The pH of the CM was adjusted to 5.80 ± 0.2 prior to autoclaving using dilute solutions of 1 N KOH and 0.02% HCl. Aliquots of the CM (4 mL) were dispensed into glass culture tubes (18 × 90 mm). Each glass culture tube was sealed with autoclavable plastic caps and autoclaved at 121 °C and a pressure of 1.1 kg/cm^2^ for 20 min. For subsequent subcultures, 125 mL glass jars containing 20 mL aliquots of CM were used. For the multiplication trial, the CM was supplemented with the corresponding plant growth regulators (PGRs) combinations (Table 5).

#### 3.3.3. Establishment of Seedlings and *In Vitro* Germination

Aseptic seeds were placed in a humid chamber on filter paper moistened with sterile distilled water. Then, they were transferred to a growth chamber at 26 ± 2 °C. To promote germination, cultures were initially maintained in total darkness for the first three days. Subsequently, they were exposed to a 16 h photoperiod, with a photosynthetic photon flux density (PPFD) of 112 μmol/m^2^.s provided by cool white, fluorescent light (Philips). Germination was monitored daily for 15 days and was considered to have occurred when the radicle length reached at least 1 mm. Successfully germinated seedlings were then transferred to glass tubes with CM. After 60 days of culture, seedling length was evaluated. binodal segments were excised and used as explants to establish shoot cultures. For the establishment of seedlings from *in vitro* seed germination, the experiments were carried out with 400 seeds in each treatment × 3 replicates.

#### 3.3.4. *In Vitro* Induction and Multiplication of Shoots

The explants obtained were cultivated in 20 mL of CM either alone or supplemented with different combinations of plant growth regulators (PGRs) (Table 5). The PGR combinations were selected based on their well-established roles in plant micropropagation and on previous reports involving *Larrea* and/or related woody species. BAP was selected as a cytokinin because of its frequent use in promoting axillary shoots and nodal development, whereas IBA was selected as an auxin because of its well-established role in root induction and development. Their combination was therefore evaluated to determine whether a balance between shoot multiplication and rooting could improve the overall propagation response of *L. cuneifolia*. Other PGRs were also evaluated individually in the present study, allowing the response to the IBA–BAP combination to be compared with those obtained with different auxins and cytokinin.

The tubes were maintained at 26 ± 2 °C under a 16 h photoperiod, with a photosynthetic photon flux density (PPFD) of 112 μmol/m^2^.s provided by cool white, fluorescent light (Philips). The cultures were kept for 60 days under the same conditions of establishment stage. Then, the regenerated shoots (at least 3 cm in length) were subcultured on the same fresh medium under the same conditions.

For shoot multiplication experiments, two independent trials were performed, with 25 replicates per biological treatment.

#### 3.3.5. *In Vitro* Rooting of Shoots

After 60 days of culture on the various CM media supplemented with PGRs (Table 5), the rooting capacity of the regenerated shoots was evaluated. Rooting was assessed as the percentage of explants that developed at least one visible root during the culture period. Thus, 100% rooting indicates that all explants evaluated in that treatment produced at least one root. Two independent experimental trials were conducted, each consisting of 25 replicates per treatment. The percentage of rooted shoots was recorded to determine the overall efficiency of the protocol.

### 3.4. Ex Vitro Acclimatization Procedure

Plantlets were carefully removed from the glass culture jars using sterile forceps, and residual culture medium adhering to the roots and basal stem parts were gently removed by rinsing with distilled water, taking care to minimize root damage. The plantlets were subsequently transferred to the acclimatization substrate. Each rooted plantlet was transplanted into plastic pots (200 mL) containing a mixture of commercial substrate containing peat moss and perlite (Growmix^®^ Multipro, Terrafertil S.A., Buenos Aires, Argentina) and washed river sand at 2:1 *v*/*v* ratio. The sand was sieved to obtain a particle size of approximately 0.5 mm and was washed and sterilized before use. Its detailed mineralogical composition was not determined. The substrate mixture was sterilized by autoclaving prior to use. The pots were placed inside transparent plastic containers, which were fully covered to maintain high relative humidity (70–85% RH range). These containers were kept in a greenhouse under controlled conditions (30 ± 2 °C temperature, 16 h photoperiod, under full-spectrum LEDs of 120 µmol/m^2^.s). To ensure adequate hydration, the plants were watered daily with tap water. A progressive decline in RH of 5% per day was implemented to ensure gradual acclimatization to *ex vitro* conditions. During this phase, the environmental conditions were carefully controlled. Survival was recorded every 30 days. As the plants grew, they were transplanted into progressively larger pots (500 mL, 1000 mL and 3000 mL) and maintained under controlled greenhouse conditions. At 11 months, one group of plants was transferred to the field, and the rest remained in the greenhouse to allow for phytochemical comparisons.

#### Field Reintroduction and Establishment of Micropropagated *L. cuneifolia* Plants

*L. cuneifolia* plants obtained by micropropagation for 11 months in a greenhouse were transferred to their natural habitat in Ampimpa, Amaicha del Valle (1960 m a.s.l.; 26°35′33″ S; 65°55′00″ W), Tucumán, Argentina. The plants were transplanted into the field to evaluate their survival and adaptation to the local environmental conditions. Periodic monitoring each 2 months was conducted for 1 year to assess the long-term success of the *in vitro* propagation and reintroduction protocol. Afterward, the number of viable plants was determined, and the phytochemical profile was compared with wild plants.

### 3.5. Phytochemical Profile of Aerial Parts of Wild Plants, Reintroduced and In Vitro Plants

#### 3.5.1. Plant Material

Leaves were randomly collected from wild adult plants and micropropagated plants maintained for 4 and 8 months in Ampimpa (Tucumán) without any selection based on leaf size or position, in order to obtain a representative sample of the foliage; subsequently, they were dried to constant weight in a forced air oven (Dhacel, FD101, Buenos Aires, Argentina) at 40 °C and then ground in a helical mill (Numak, F 100 Power ½ hp-0.75 KW, Brusque, Brazil). Particle size, or granulometry, was determined by vibrating screen sieving (Zonytest, Buenos Aires, Argentina) with 840, 500, 149, 105, and 74 µm sieves.

The dried and milled plant material was vacuum-packed in seven-layer polyamide/polyethylene (PA/PE) bags (Terlizzi, Buenos Aires, Argentina) to minimize exposure to oxygen and moisture and to protect the samples from physical and microbial contamination during storage. The bags were stored at room temperature.

#### 3.5.2. Ethanolic Extracts Preparation of *L. cuneifolia*

The powdered plant material (leaves) was macerated in 60% (*v*/*v*) ethanol at a ratio of 1 g of powder to 20 mL of solvent for 60 min (comprising six cycles of 10 min of operation and 2 min of pause) using ultrasound assistance, at a power of 70 W and a frequency of 26 kHz, in a 200 mL bath at 40 °C (Ultrasonic Washer Arcano Model PS-10A, Shandong Arcano Ultrasonic Technology Co., Ltd., Jinan, China). The extracts were then vacuum-filtered (Cellulose filters, 6–15 µm) and dried in a vacuum oven (Arcano DF2-6020, Scientific Dominguez, Tucumán, Argentina), at 40 °C until they reached a constant weight. The yield of soluble principle (SP) was determined by gravimetric methods (Analytical Balance OHAUS Model PA-214, Parsippany, NJ, USA), and the results were expressed as g of soluble principle in 60% ethanol per g of dry plant material (PM). All extracts were obtained in biological triplicate (powder from different plants, independent extractions).

#### 3.5.3. Foliar Surface Washing with Chloroform

Wild, reintroduced and micropropagated plant leaves were briefly immersed in chloroform for 20 s to extract surface (epicuticular) metabolites at 24 °C. The resulting extracts were filtered under vacuum (cellulose filters, 6–15 µm) and evaporated to dryness in a rotary evaporator (Büchi Labortechnik AG R-110, Flawil, Switzerland), and the residues were subsequently dissolved in ethanol. The yield of the extracted metabolites was determined.

#### 3.5.4. Total Phenolic Compounds and Total Flavonoid Content Determination

The total content of phenolic compounds of all extracts was estimated by using Folin–Ciocalteu reagent [53]. The blue color developed was read at 765 nm in a UV/visible spectrophotometer (Jasco V-630, Thermo Fisher Scientific, Tokyo, Japan). It should be noted that the Folin–Ciocalteu assay provides an estimate of total phenolic content based on reducing capacity and is not specific to phenolic compounds; therefore, other reducing constituents present in the extracts may contribute to the measured values. Total flavonoids were estimated by using the Woisky and Salatino method with AlCl_3_ reagent [54]. The determinations were performed in triplicate, and the results were expressed as μg of gallic acid equivalents (GAE) per milliliter of extract (μg GAE/mL) and quercetin equivalents (QE) per milliliter of extract (μg QE/mL) for polyphenols and flavonoids, respectively.

#### 3.5.5. Identification of the Compounds in *L. cuneifolia* Extracts by HPLC Coupled to an Orbitrap Exploris 240 Mass Spectrometer

The separation and tentative identification of the compounds of *L. cuneifolia* extracts were performed using a HPLC system (Thermo Fisher Scientific, Bremen, Germany) equipped with binary pump (Dionex Softron GmbH-VCP12A) and automatic sampling (Dionex Softron GmbH-VCA12A) coupled to an Orbitrap Exploris 240 high resolution mass spectrometer (Thermo Fisher Scientific) equipped with an OptaMax NG™ heated electrospray ionization (H-ESI) source. The dried extract was dissolved in HPLC-grade ethanol at a concentration of 1 mg/mL. Chromatographic separation was performed using an Accucore™-aQ C18 column (150 mm × 2.1 mm, 2.6 μm) equipped with the corresponding guard column, at a constant flow rate of 0.2 mL/min for a total analysis time of 30 min. The column compartment and autosampler were maintained at 25 °C, using an injection volume of 2.3 μL. The mobile phase consisted of a binary mixture of H_2_O with 0.1% (*v*/*v*) formic acid (FA) (Solvent A) and acetonitrile containing 0.1% (*v*/*v*) FA (Solvent B). The following gradient was applied: 30.0% to 40.0% B from 0.00–10.94 min, 40.0% to 45.0% B from 10.94–15.63 min, 45.0% to 70.0% B from 15.63 to 21.88 min, 70.0–100.0% B from 21.88 to 25.01 min, and 100.0% to 30.0% B from 25.01 to 30.00 min. Instrument control and data acquisition were performed using Chromeleon software (version 7.3.1; Thermo Fisher Scientific).

Mass spectrometric analysis was performed in negative-ion H-ESI mode using a static spray voltage of 2.5 kV. The ion transfer tube and vaporizer temperatures were maintained at 320 and 275 °C, respectively. Full-scan high-resolution mass spectra were acquired at a resolution of 240,000 over an m/z range of 100–1000. Data-dependent MS/MS (ddMS^2^) acquisition was performed on the 20 most intense precursor ions exceeding an intensity threshold of 5.0 × 10^3^. Precursor ions were fragmented by higher-energy collisional dissociation (HCD) using a normalized collision energy of 35%, and MS/MS spectra were acquired in the Orbitrap at a resolution of 15,000.

#### 3.5.6. Nordihydroguaiaretic Acid Quantification by HPLC-DAD

*L. cuneifolia* extracts were analyzed by means of high-performance liquid chromatography (HPLC), using a system including a Waters 1525 binary pump and a manual injection valve (Rheodyne Inc., Cotati, CA, USA), with a 20 µL loop. The separation was performed on a Waters C18, 5 µm, 4.6 × 250 mm column, kept at 25 °C. Analyses were performed by using a linear gradient composed of 1% formic acid in water (Solvent A) and acetonitrile (Solvent B) as follows: 30% to 40% B over 35 min, increasing to 45% B at 50 min, 70% B at 70 min, 70% to 100% B from 70 to 80 min, 100% to 30% B from 80 to 85 min, and keeping the 30% until the end at 95 min. The flow rate was 0.5 mL/min and the injected volume was 20 µL. Compounds were monitored at 280 nm. Compounds present in the extracts were identified by comparing retention times and UV-visible profiles with those of commercial reference compounds. Quantification of the different compounds was performed as NDGA equivalent at 280 nm.

### 3.6. Biological Activities of Plant Extracts: Antioxidant and Anti-Inflammatory Activity

Free radical scavenging capacity by the ABTS^•+^ method of extracts obtained from micropropagated plants and wild plants was determined according to Re et al. (1999) [55]. ABTS^•+^ was generated by reacting ABTS^•+^ (7 mM) with potassium persulfate (2.45 mM) and allowing the mixture to stand in the dark at room temperature for 16 h. Before use, the ABTS^•+^ solution was diluted with ethanol to obtain an absorbance of 0.9 at 734 nm. Different concentrations of extract were mixed with ABTS^•+^ and maintained for 6 min at room temperature. The product was determined by recording the absorbance at 734 nm in a microplate reader (Multiskan GO, Thermo Fisher Scientific, Vantaa, Finland). The results were expressed in terms of 50% free radical scavenging concentration (SC_50_), which is defined as the µg GAE/mL required to eliminate 50% of free radicals. The SC_50_ was expressed in µg GAE/mL.

The H_2_O_2_ scavenging was assessed by Fernando and Soysa [56]. Different concentrations of the extract were mixed with H_2_O_2_ (0.7 mM), allowing it to stand for 3 min at room temperature. Then, phenol (12 mM), 4-aminoantipyrene (0.5 mM), and Horseradish peroxidase (1.0 U/mL) were added. It was incubated for 30 min at 37 °C, and the product was determined by recording the absorbance at 504 nm in a microplate reader (Multiskan GO, Thermo Fisher Scientific, Vantaa, Finland). Results are expressed as SC_50_ values (μg GAE/mL).

The effect of different concentrations of extracts on the activity of xanthine-oxidase (XO) (0.003 U) was determined at 290 nm (Multiskan GO, Thermo Fisher Scientific, Vantaa, Finland) by measuring the synthesis of uric acid from xanthine as substrate (1 mM) [57]. The reaction mixture was incubated at 25 °C for 30 min. The IC_50_ was defined as the concentration of inhibitor required to reduce enzyme activity to 50% of the activity measured in the absence of inhibitor. The inhibitory concentration of 50 (IC_50_) of enzyme activity was expressed in μg GAE/mL.

### 3.7. Statistical Analysis

Statistical analyses were performed using R (version 4.3.3) [58]. Morphogenic responses of the explants were evaluated considering the number of leaves, number of nodes, and the presence or absence of roots and calluses as response variables. Descriptive results for the number of leaves and number of nodes are expressed as mean ± standard deviation (SD). Treatment effects on these variables were assessed using linear mixed-effects models for repeated measures. The significance of fixed effects was determined by analysis of variance (ANOVA) on the fitted models, and treatments were compared pairwise using Tukey’s test (α = 0.05), with results grouped using compact letter representations. Rooting (presence/absence) was analyzed using a generalized linear model with a binomial distribution and a logit link function, with treatment as the explanatory factor. Overall treatment significance was assessed by analysis of deviance using chi-square tests. Pairwise comparisons among treatments were performed using estimated marginal means with Tukey adjustment (α = 0.05) on the logit scale, and results were grouped using compact letter representations.

To simultaneously integrate morphogenic variables and explore general response patterns among treatments, a principal component analysis (PCA) was performed on the variables recorded at the final evaluation (60 days) number of leaves, number of nodes and presence of roots, callus and shoots. Principal component analysis (PCA) was performed on the phytochemical content and antioxidant activity to identify the most effective solvent systems.

## 4. Conclusions

The present study demonstrates, for the first time, that micropropagation of *Larrea cuneifolia* not only enables efficient clonal propagation but also preserves the phytochemical and biological characteristics of the species. During greenhouse acclimatization and subsequent field establishment, plants progressively recovered their capacity to synthesize phenolic compounds and NDGA while maintaining the characteristic phytochemical fingerprint of the genus. Together with the seasonal and circadian metabolic plasticity recently described for wild populations of *L. cuneifolia*, the present results demonstrate that the species retains a remarkable capacity to modulate its specialized metabolism after micropropagation and field establishment while preserving its characteristic phytochemical identity. Furthermore, the antioxidant and anti-inflammatory properties of acclimatized plants were comparable to those of wild individuals, indicating that *in vitro* propagation does not compromise the production of bioactive metabolites. Collectively, these findings demonstrate that the developed protocol represents a valuable strategy for the conservation of *L. cuneifolia* and provides a sustainable source of phytochemically standardized plant material with potential pharmaceutical or cosmetic applications. In the future, further studies will be conducted to determine the contribution of each compound, as well as the possible additive or synergistic interactions between phenolic compounds, flavonoids, lignans, and other components—such as terpenes—in antioxidant and anti-inflammatory activity. Undoubtedly, a deeper understanding of the role of the phytochemicals of *L. cuneifolia* in the molecular mechanisms related to inflammatory and oxidative processes is needed.

## Figures and Tables

**Figure 1:**
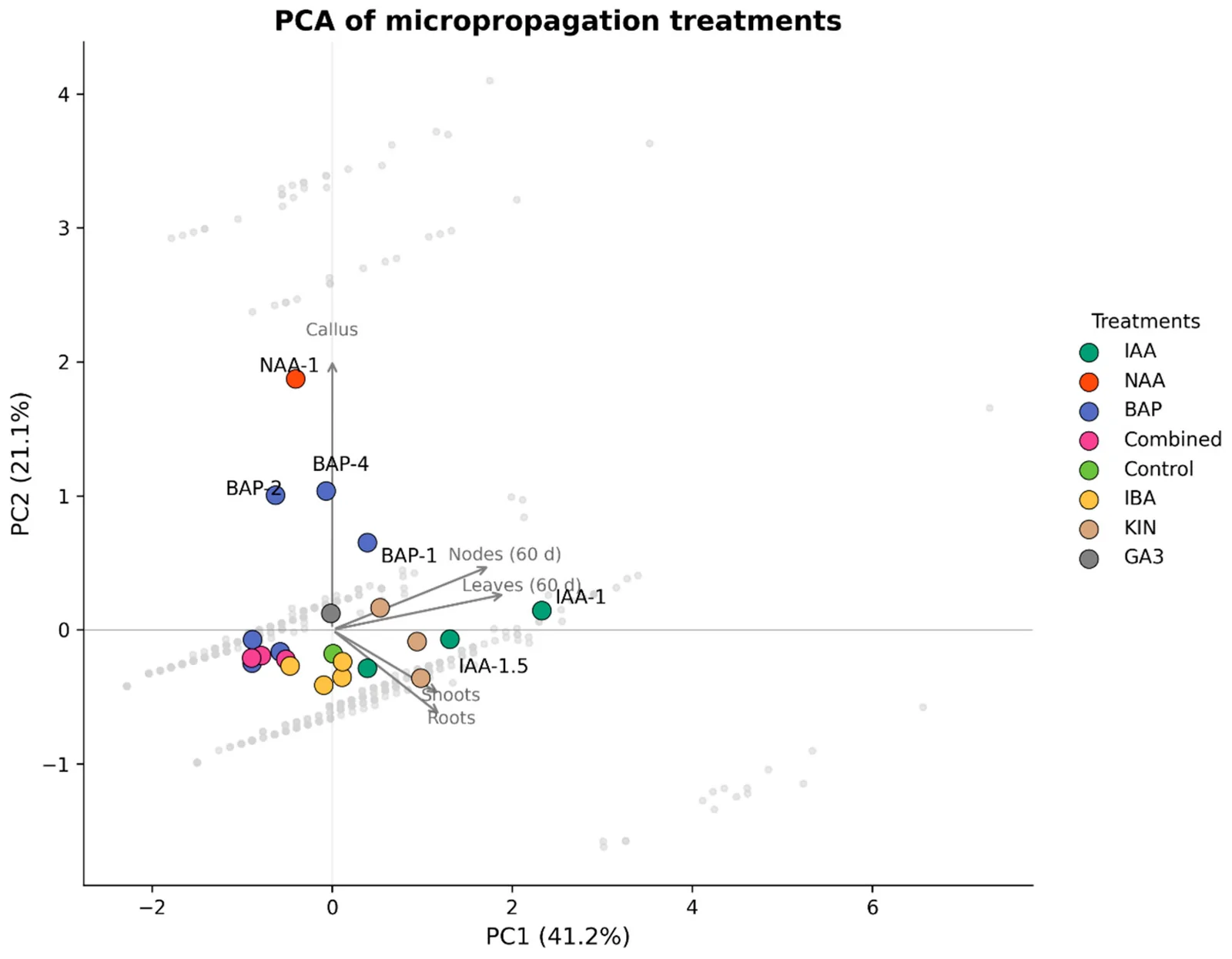
Principal Component Analysis (PCA) of the response to different PGRs used to evaluate the *in vitro* micropropagation of *L. cuneifolia.* IAA, indole-3-acetic acid; GA_3_, gibberellic acid; BAP, 6-benzylaminopurine; IBA, indole-3-butyric acid; KIN, kinetin; NAA: 1-naphthaleneacetic acid. IAA-1: Sample IAA-1 contained 1 mg/L of indole-3-acetic acid in growth media; BAP-1: Sample BAP-1 contained 1 mg/L of 6-benzylaminopurine in growth media.

**Figure 2:**
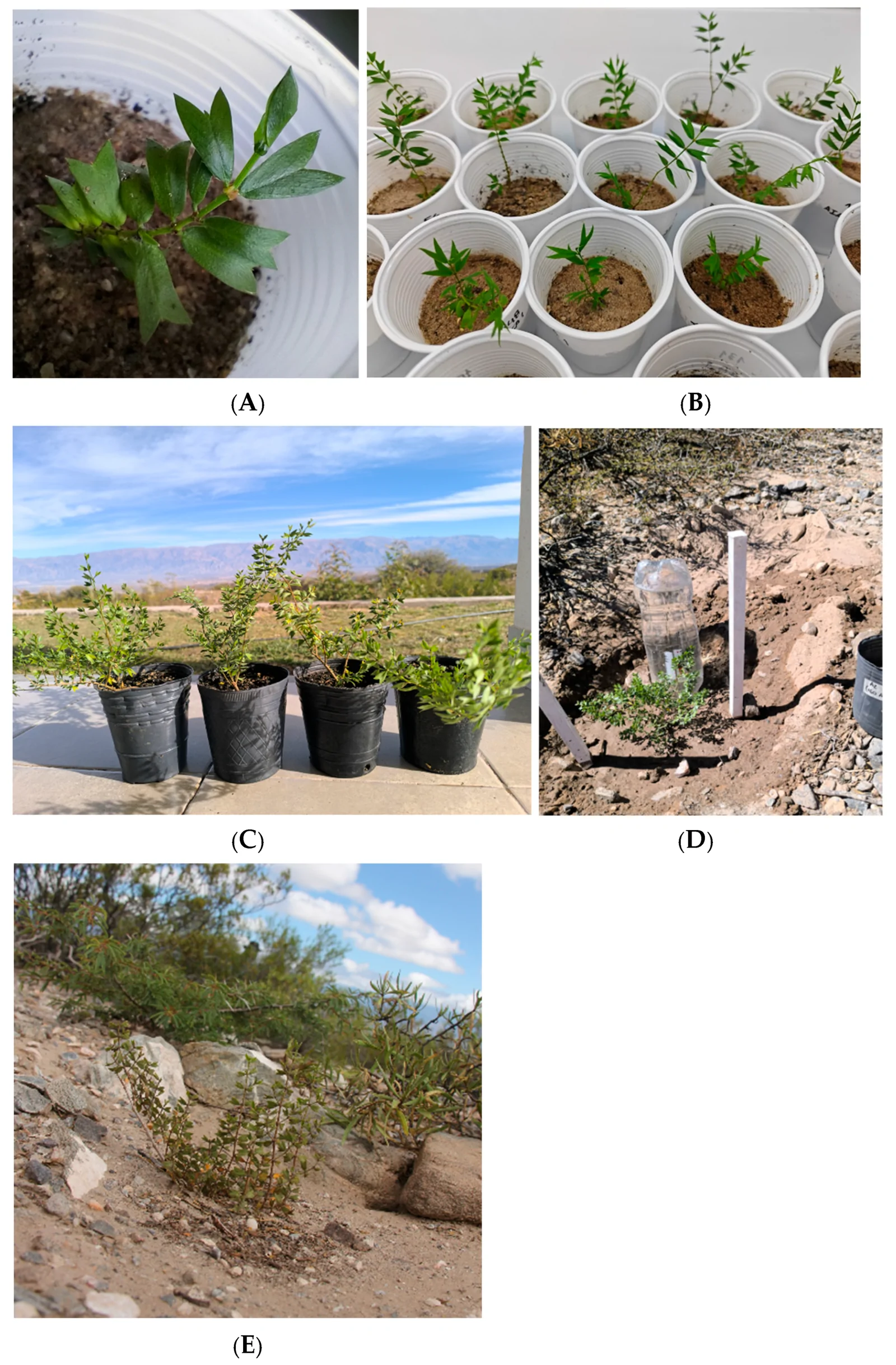
Acclimatization of micropropagated plants of *L. cuneifolia* in Ampimpa locality, at 1960 m a.s.l. in Valles Calchaquíes (Argentina): (**A**) Plant transplanted into substrate. (**B**) Plants after one month of acclimatization in the greenhouse. (**C**) Plants before being transplanted to Ampimpa at 1960 m a.s.l. (**D**) Plant already transplanted into its habitat (Ampimpa at 1960 m a.s.l.). (**E**) Plant in Ampimpa four months after transplanting.

**Figure 3:**
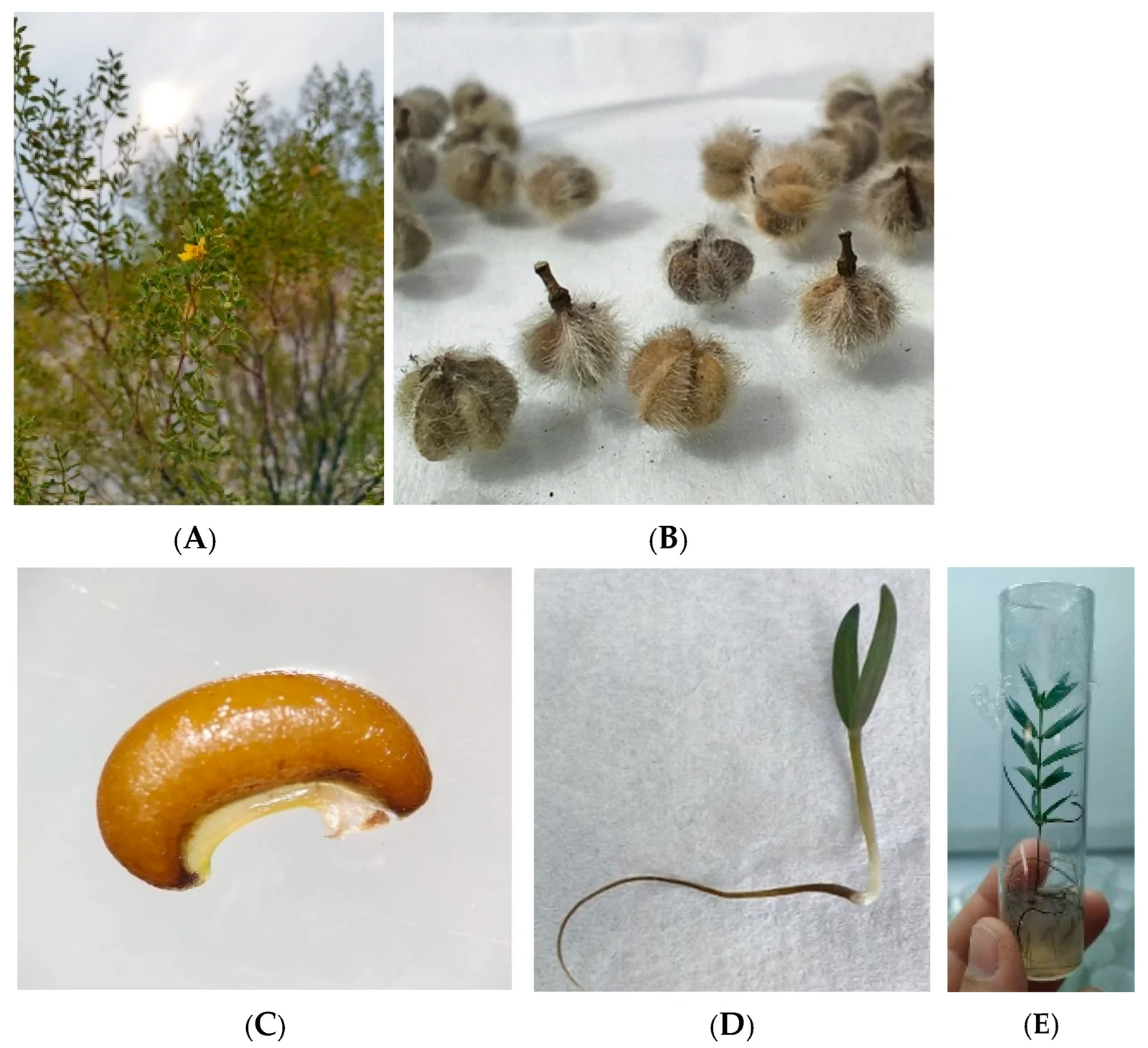
(**A**) *L. cuneifolia* in Ampimpa (Tucumán, Argentina). (**B**) Mature fruits. (**C**) Seeds. (**D**) *In vitro* plantlet. (**E**) Production of axenic explants through *in vitro* seed germination.

**Table 1:** Effect of different concentrations of growth regulators on the number of leaves, nodes, and rooting percentage obtained by *in vitro* culture of *L. cuneifolia* at 60 days of culture.

Treatment	Average N^o^. of Leaves/Explant Mean ± SD	Average N^o^. of Nodes/Explant Mean ± SD	Rooting (%)
IAA-1	14.44 ± 5.12 ^e^	7.38 ± 3.56 ^b^	62.50 ^abc^
KIN-1	12.38 ± 4.24 ^de^	3.06 ± 1.29 ^a^	68.75 ^abc^
KIN-0.5	12.00 ± 3.04 ^cde^	3.82 ± 2.01 ^a^	76.47 ^bc^
IAA-1.5	11.93 ± 6.68 ^bcde^	3.13 ± 1.96 ^a^	80.00 ^bc^
KIN-0.25	11.41 ± 4.95 ^abcde^	3.59 ± 2.06 ^a^	88.24 ^bc^
BAP-1	10.89 ± 3.83 ^abcde^	3.89 ± 1.57 ^a^	33.33 ^abc^
GA_3_-1	10.58 ± 4.17 ^abcde^	3.67 ± 1.97 ^a^	7.69 ^abc^
IAA-0.5	10.43 ± 6.06 ^abcde^	3.21 ± 2.61 ^a^	57.14 ^abc^
BAP-4	10.08 ± 4.01 ^abcde^	3.17 ± 2.33 ^a^	25.00 ^abc^
IBA-1	8.49 ± 4.02 ^abcd^	3.13 ± 2.04 ^a^	74.47 ^c^
IBA-0.5	8.45 ± 4.93 ^abcd^	2.38 ± 1.27 ^a^	72.41 ^bc^
CONTROL	8.27 ± 4.64 ^abcd^	2.84 ± 2.30 ^a^	74.19 ^c^
IBA-1.5	8.06 ± 6.74 ^abcd^	3.75 ± 3.30 ^a^	62.50 ^abc^
IBA-0.1/BAP-0.25	8.00 ± 4.47 ^abcd^	2.13 ± 1.19 ^a^	33.33 ^abc^
IBA-0.1/BAP-0.125	7.27 ± 4.50 ^abcd^	1.80 ± 1.26 ^a^	20.00 ^abc^
IBA-0.1	7.25 ± 3.88 ^abcd^	2.39 ± 1.69 ^a^	42.86 ^abc^
BAP-0.125	7.16 ± 3.69 ^abcd^	2.53 ± 1.22 ^a^	26.32 ^abc^
IBA-0.1/BAP-0.5	6.82 ± 3.54 ^abcd^	1.64 ± 0.50 ^a^	18.18 ^abc^
NAA-1	6.73 ± 3.26 ^abc^	2.09 ± 2.24 ^a^	68.18 ^abc^
BAP-0.5	6.48 ± 3.31 ^ab^	2.32 ± 1.28 ^a^	4.00 ^a^
BAP-0.25	6.44 ± 4.74 ^a^	1.68 ± 1.11 ^a^	24.00 ^ab^
BAP-2	5.55 ± 4.72 ^abc^	3.09 ± 1.70 ^a^	27.27 ^abc^

IAA: indole-3-acetic acid; GA_3_: gibberellic acid; BAP: 6-benzylaminopurine; IBA: indole-3-butyric acid; KIN: kinetin; NAA: 1-naphthaleneacetic acid. The number next to the abbreviation for each growth promoter indicates its concentration in mg/L; for example, IAA-1: Sample IAA-1 contained 1 mg/L of indole-3-acetic acid in growth media; BAP-1: Sample BAP-1 contained 1 mg/L of 6-benzylaminopurine in growth media; SD: Standard deviation. The different letters show significant differences between the different PGRs treatments according to Tukey’s multiple comparisons test (*p* ≤ 0.05).

**Table 2:** Comparative phytochemical characterization of *in vitro* micropropagated, *ex vitro*-acclimatized, and wild *L. cuneifolia* aerial parts.

Extracts of *L. cuneifolia* Leaves	Total Phenolic Compounds	Phenolic Compounds Removed by Foliar Washed	Flavonoid Compounds	Flavonoid Removed by Foliar Washed %
	µg GAE/mL	%	µg QE/mL	
MP-2	2440 ± 96		610 ± 18	
MP-2+8G	3140 ± 22		750 ± 21	
MP-2+8G Foliar washed	907 ± 12	28.9	104 ± 5	14.0
MP-2+11G	7020 ± 280		550 ± 41	
MP-2+11G Foliar washed	3430 ± 160	48.9	370 ± 32	67.7
MP-2+15G	8790 ± 290		547 ± 8	
MP-2+15G Foliar washed	4440 ± 550	50.5	370 ± 30	68.0
MP-2+11G+4F	7370 ± 200		548 ± 12	
MP-2+11G+4F Foliar washed	3020 ± 34	40.9	126.6 ± 1.6	23
YWP	7190 ± 350		475 ± 15	
YWP Foliar washed	1320 ± 80	18.4	43.9 ± 1.8	10.3
AWP	7800 ± 450		421 ± 12	
AWP Foliar washed	1420 ± 100	18.1	54.4 ± 2.3	12.9

µg GAE/mL: micrograms of gallic acid equivalent per milliliter. µg QE/mL: micrograms of quercetin equivalent per milliliter. Total phenolic and flavonoid contents were determined in leaf extracts. Leaf surface phenolics and flavonoids correspond to the epicuticular resin recovered by washing of leaf surface with chloroform (Foliar washed). MP-2: extract of two-month-old micropropagated plants; MP-2+8G: extract of two-month-old micropropagated plants and maintained in greenhouse for 8 months; MP-2+11G: extracts of two-month-old micropropagated plants and maintained in greenhouse for 11 months; MP-2+15G: extract of two-month-old micropropagated plants and maintained in greenhouse for 15 months; MP-2+11G+4F: extract of two-month-old micropropagated plants and maintained in greenhouse for 11 months and for 4 months in field; YWP: extract of Young Wild Plant collected in Ampimpa at 1960 m a.s.l.; AWP: extract of Adult Wild Plant collected in Ampimpa at 1960 m a.s.l.

**Table 3:** Level of NDGA in leaves of micropropagated and *ex vitro* acclimatization *L. cuneifolia.*

Samples Leaves	mg NDGA/mL	mg NDGA/g DW
MP-2	0.40 ± 0.1	8.00 ± 0.04
MP-2+8G	3.34 ± 0.3	66.81 ± 0.05
MP-2+15G	5.06 ± 0.2	101.20 ± 0.03
MP-2+11G+4F	5.61 ± 0.2	112.20 ± 0.04
YWP	4.73 ± 0.3	94.60 ± 0.06

NDGA: nordihydroguaiaretic acid, DW: Dry weight of extract of leaves. MP-2: Two-month-old micropropagated plants; MP-2+8G: Two-month-old micropropagated plants and maintained in greenhouse for 8 months; MP-2+15G: Two-month-old micropropagated plants and maintained in greenhouse for 15 months; MP-2+11G+4F: Two-month-old micropropagated plants and maintained in greenhouse for 11 months and for 4 months in Ampimpa at 1960 m a.s.l.; YWP: Young Wild Plant collected in Ampimpa at 1960 m a.s.l.

**Table 4:** Antioxidant and anti-inflammatory activities of extracts obtained from plant material of *L. cuneifolia.*

Samples	ABTS ^•+^ SC_50_ (µg GAE/mL)	H_2_O_2_ SC_50_ (µg GAE/mL)	XO IC_50_ (µg GAE/mL)
MP-2+15G	3.96 ± 0.08	20.8 ± 2.00	3.41 ± 0.30
MP-2+11G+4F	4.56 ± 0.12	17.15 ± 1.00	4.06 ± 0.34
YWP	4.90 ± 0.09	15.67 ± 1.00	4.17 ± 0.37

µg GAE/mL: micrograms of gallic acid equivalent per milliliter. ABTS^•+^, 2,2′-azino-bis(3- ethylbenzothiazoline-6-sulfonic acid) diammonium salt; H_2_O_2_, hydrogen peroxide; XO, xanthine oxidase. SC_50_: scavenging concentration necessary to remove 50% of free radicals. IC_50_: inhibitory concentration of 50% of enzyme activity. MP-2: Two-month-old micropropagated plants; MP-2+15G: Two-month-old micropropagated plants and maintained in greenhouse for 15 months; MP-2+11G+4F: Two-month-old micropropagated plants and maintained in greenhouse for 11 months and for 4 months in field; YWP: Young Wild Plant collected in Ampimpa at 1960 m a.s.l.

**Table 5:** Different combinations of plant growth regulators (PGRs).

PGRs	(mg/L)	PGRs	(mg/L)
Control			
GA_3_	1		
IAA	0.5		
IAA	1		
IAA	1.5		
NAA	1		
BAP	0.125		
BAP	0.25		
BAP	0.5		
BAP	1		
BAP	2		
BAP	4		
IBA	0.1	BAP	0.125
IBA	0.1	BAP	0.25
IBA	0.1	BAP	0.5
IBA	0.1		
IBA	0.5		
IBA	1		
IBA	1.5		
KIN	0.25		
KIN	0.5		
KIN	1		

PGRs, plant growth regulators; IAA, indole-3-acetic acid; GA_3_, gibberellic acid; BAP, 6-benzylaminopurine; IBA, indole-3-butyric acid; KIN, kinetin, NAA, 1-naphthalene-acetic acid.

## Data Availability

The original contributions presented in this study are included in the article/Supplementary Material. Further inquiries can be directed to the corresponding author.

## Supplementary Materials

The following supporting information can be downloaded at: https://www.mdpi.com/article/10.3390/plants15213323/s1, Figure S1: HPLC–HESI–Orbitrap-HRMS/MS chromatograms of (A) wild *Larrea cuneifolia* extract (Base peak, negative mode) and (B)—Micropropagated *Larrea cuneifolia* extract (Base peak, negative mode); Table S1: HPLC–HESI–Orbitrap–HR MS/MS analyses of wild and micropropagated *L. cuneifolia* extracts (A) Micropropagated *L. cuneifolia* and reintroduced in Valles Calchaquíes (B) Wild *L. cuneifolia* from Valles Calchaquíes (C) Foliar washes.
