# Supplementary material for: Phytochemical Profile and Antioxidant and Anti-Inflammatory Properties of Larrea cuneifolia, a Medicinal Plant of Argentina, Obtained by Micropropagation, Acclimatized, and Established in the Field, Compared with Wild Plants
Source: Plants (Basel). 2026 Oct 27;15(21):3323. doi: 10.3390/plants15213323 (PMC13766147; doi:10.3390/plants15213323)
Supplement: Supplementary file 1 [file plants-15-03323-s001.zip › plants-4538565-supplementary.pdf]

Table S1. HPLC–HESI–Orbitrap–HR MS/MS analyses of wild and micropropagated *L. cuneifolia* extracts A) Micropropagated *L. cuneifolia* and reintroduced in Valles Calchaquíes B) Wild *L. cuneifolia* from Valles Calchaquíes C) Foliar washes

CODE	RT [min]	Name	Molecular Formula	Measured ion (m/z) [M-H]-	Accuracy (ppm)	A	B	C	Type compounds
1	1.05	Xylitol	$C_5H_{12}O_5$	151.06119	-0.23		X		Polyalcohol
2	1.06	D-Pinitol	$C_7H_{14}O_6$	193.07168	-0.44		X		Polyol cyclic
3	1.06	Dihydroquercetin (Taxifolin)	$C_{15}H_{12}O_7$	303.05106	0.10	X			Flavonoid
4	1.08	D-(+)-Malic acid	$C_4H_6O_5$	133.01424	0.38	X	X		Tricarboxylic acid
5	1.09	Fumaric acid	$C_4H_4O_4$	115.00373	-0.25	X	X		Tricarboxylic acid
6	1.07	D-(-) Quinic acid	$C_7H_{12}O_6$	191.05605	-0.38	X	X		Phenolic acid
7	1.13	Fukiic acid	$C_{11}H_{12}O_8$	271.04590	-0.16	X	X		Phenolic acid
8	1.13	Quercetin-3-O-rutinoside	$C_{27}H_{30}O_{16}$	593.15106	-0.27	X	X		Flavonoid
9	1.14	Chlorogenic acid	$C_{16}H_{18}O_9$	353.08752	-0.66	X	X		Phenolic acid
10	1.14	Quercetin-3-β-D-glucoside	$C_{21}H_{20}O_{12}$	463.08786	-0.73	X	X		Flavonoid
11	1.15	Isorhamnetin-3-O-rutinoside (Narcisine).	$C_{28}H_{32}O_{16}$	623.16147	-0.47		X		Flavonoid
12	1.16	3,3′,4',5,5',7 hexahydroxy flavone (Myricetin)	$C_{15}H_{10}O_8$	317.03018	-0.34		X		Flavonoid
13	1.17	Catechin	$C_{15}H_{14}O_6$	289.07182	0.22	X			Flavonoid
14	1.22	Hesperetin 3'-O-glucuronide	$C_{22}H_{22}O_{12}$	477.10388	0.07	X			Flavonoid
15	1.26	4-methyl catechol	$C_7H_8O_2$	123.04513	-0.30		X		Phenolic acid
16	1.27	4-Hydroxy-3-methoxy cinnamic acid (Ferulic acid)	$C_{10}H_{10}O_4$	193.05062	-0.07		X		Phenolic acid
17	1.29	4-hydroxy benzoic acid	$C_7H_6O_3$	137.02444	0.20		X		Phenolic acid
18	1.31	Kaempferol-3-0-glucoside	$C_{22}H_{22}O_{11}$	461.10892	-0.04		X		Flavonoid
19	1.36	Chalcone	$C_{27}H_{30}O_6$	449.19696	-0.01		X		Chalcone
20	1.41	3,4 Dihydroxy hydro cinnamic acid (hydro-caffeic acid)	$C_9H_{10}O_4$	181.05063	-0.02	X	X		Phenolic acid
21	1.51	3',4',5'-trimethoxyflavone (Jacein)	$C_{18}H_{16}O_5$	311.09256	0.18		X		Flavonoid
22	1.54	3'-O-methyl quercetin	$C_{16}H_{12}O_7$	315.06659	0.09	X	X		Flavonoid
23	1.55	Epicatechin	$C_{15}H_{14}O_6$	289.07167	-0.31		X		Flavanol
24	1.69	2,3 dihydroluteolin (Eriodictyol)	$C_{15}H_{12}O_6$	287.05610	-0.04	X	X		Flavonoid
25	1.75	5,7,3',4'-tetrahydroxy-3-Methoxyflavone	$C_{16}H_{12}O_7$	315.05118	0.12	X	X		Flavonoid
26	1.78	Kaempferol	$C_{15}H_{10}O_6$	285.04043	-0.12		X		Flavonoid
27	1.88	4′,5,7-trimethoxyflavone	$C_{18}H_{16}O_5$	311.09242	-0.27	X	X		
28	3.21	3',5,7-Trihydroxy-4'-Methoxyflavanone (Hesperetin)	$C_{16}H_{14}O_6$	301.07187	0.19		X		Flavonoid
29	3.41	4',5,7-trihydroxy-6-methoxyflavone (Hispidulin)	$C_{16}H_{12}O_6$	299.05619	0.27	X	X	X	Flavonoid
30	4.02	3,4′,5-trihydroxy-7-Methoxyflavona (Rhamnocitrin)	$C_{16}H_{12}O_6$	299.05603	-0.27		X		Flavonoid

31	4.88	7,3' dimethyl quercetin	$C_{17}H_{14}O_7$	329.06669	0.29	X	X		Flavonoid
32	7.11	p-Hydroxyphenethyl trans-ferulate	$C_{18}H_{18}O_5$	313.10808	-0.21		X		Phenolic acid
33	7.20	3,3',4'-trihydroxy-4 methoxy 7,7'-epoxilignan	$C_{19}H_{22}O_5$	329.13940	-0.16	X	X	X	Epoxy lignan
34	7.25	3,4,3',4'-tetrahydroxy-6,7'-cyclolignan isomer	$C_{18}H_{20}O_4$	299.12885	-0.62	X	X	X	Cyclo-lignan
35	7.75	Nordihydro-guaiaretic acid (NDGA)	$C_{18}H_{22}O_4$	301.14459	-1.21	X	X	X	Lignan
36	8.88	3, 3' dihydroxy-4'-methoxy-7epoxylignan	$C_{19}H_{22}O_5$	330.3800	0.09	X		X	Lignan
37	10.33	Coniferyl ferulate	$C_{20}H_{20}O_6$	355.11865	-0.17		X		Alcohol phenylpropanoid
38	11.52	4-[4-(4-hydroxy-phenyl)-2,3 dimethyl 1-butyl-benzene-1,2 diol (Demethyl-NDGA)	$C_{18}H_{22}O_3$	285.14963	-0.35	X	X		Lignan
39	12.01	3-O-Methyl nordihydro-guaiaretic acid	$C_{19}H_{24}O_4$	315.15995	-0.73	X	X	X	Lignan
40	12.02	3,4,5,4'-Tetramethoxy-stilbene	$C_{18}H_{20}O_4$	299.12892	0.59	X	X		Stilbene
41	12.28	nordihydro-guaiaretic acid isomer 1	$C_{18}H_{22}O_4$	301.14453	0.40	X	X	X	Lignan
42	12.91	nordihydro- guaiaretic acid isomer 2	$C_{18}H_{22}O_4$	301.14453	0.00	X			Lignan
43	13.08	Kaempferol 3,7-dimethyl ether (Jaranol)	$C_{17}H_{14}O_6$	313.07173	-0.11	X	X		Flavonoid
44	13.13	nordihydro-guaiaretic acid isomer 3	$C_{18}H_{22}O_4$	301.14453	0.30	X	X	X	Lignan
45	17.01	3-O-Methyl nordihydro-guaiaretic acid	$C_{19}H_{24}O_4$	315.15995	-0.51	X	X	X	Lignan

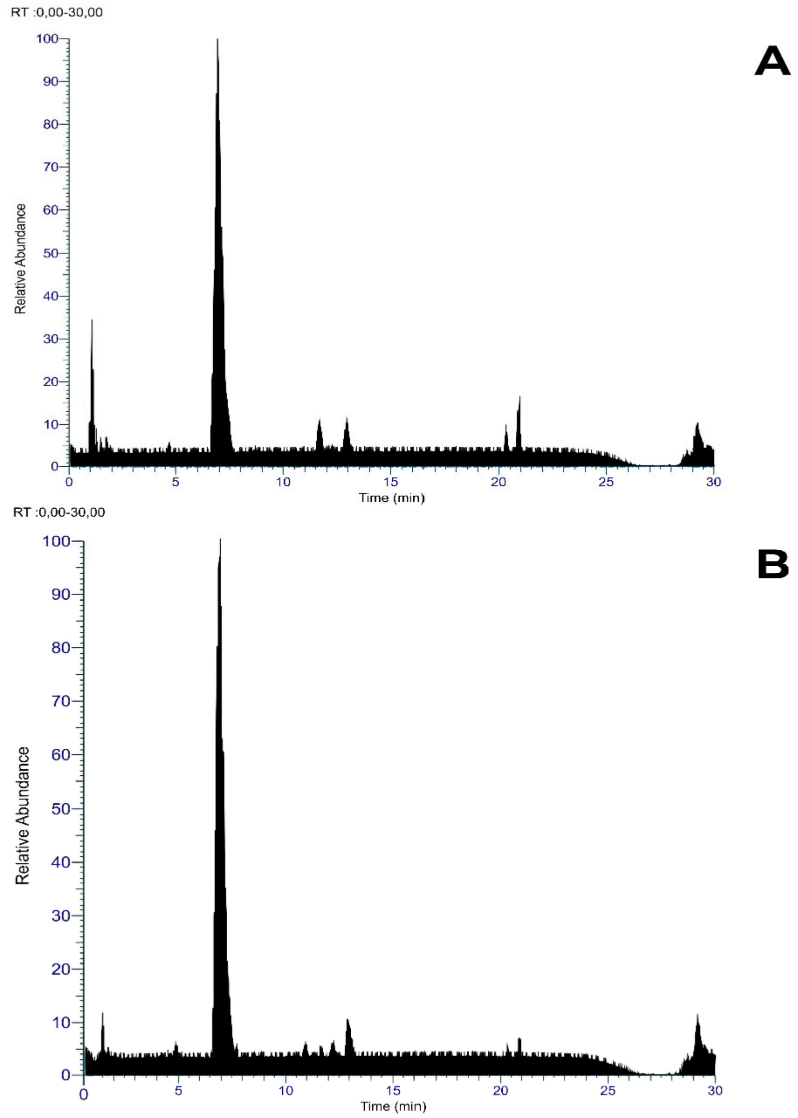

Figure S1. HPLC–HESI–Orbitrap-HRMS/MS chromatograms of A) wild *Larrea cuneifolia* extract (Base peak, negative mode) and B—Micropropagated *Larrea cuneifolia* extract (Base peak, negative mode)
